# Advances in Targeted Autophagy Modulation Strategies to Treat Cancer and Associated Treatment-Induced Cardiotoxicity

**DOI:** 10.3390/ph18050671

**Published:** 2025-05-01

**Authors:** Lauren A. Ling, Asma Boukhalfa, Andrew H. Kung, Vicky K. Yang, Howard H. Chen

**Affiliations:** 1 Molecular Cardiology Research Institute, Tufts Medical Center, 800 Washington Street, #80, Boston, MA 02111, USA; lauren.ling@tufts.edu (L.A.L.); asma.boukhlfa@gmail.com (A.B.);; 2 School of Medicine, Tufts University, 145 Harrison Avenue, Boston, MA 02111, USA; 3 Cummings School of Veterinary Medicine, Tufts University, 200 Westboro Rd., North Grafton, MA 01536, USA; vicky.yang@tufts.edu

**Keywords:** autophagy, apoptosis, cardio-oncology, cardioprotection, nanotechnology

## Abstract

Autophagy, an evolutionarily conserved process, plays an important role in cellular homeostasis and human diseases. Cardiovascular dysfunction, which presents during cancer treatment or in cancer-free individuals years after treatment, is a growing clinical challenge. Millions of cancer survivors and patients face an unpredictable risk of developing cardiotoxicity. Cardiotoxicity due to cancer treatment, as well as cancer progression, has been linked to autophagy dysregulation. Modulating autophagy has been further proposed as a therapeutic treatment for both cancer and cardiovascular disorders. The safe and effective use of autophagy modulation as a cardioprotective strategy during cancer treatment especially requires careful consideration and experimentation to minimize the impact on cancer treatment. We focus here on recent advances in targeted autophagy modulation strategies that utilize interdisciplinary approaches in biomedical sciences and are potentially translatable to treat cardiotoxicity and improve cancer treatment outcomes. This review highlights non-small molecule autophagy modulators to enhance targeted therapy, nanomedicine for autophagy modulation and monitoring, and in vitro models and future experiments needed to bring novel autophagy discoveries from basic research to clinical translation.

## 1. Background

### 1.1. Cardio-Oncology: Cardiovascular Disease Due to Cancer Treatment

Cancer therapies, including chemotherapeutics, targeted drugs, and radiation, have all been indicated to have unwanted side effects affecting major organ systems. In the cardiovascular system, toxicity from cancer therapies can cause systolic dysfunction, hypertension, arrhythmia, myocarditis, heart failure, and in some cases, death [1]. For instance, in women 50 years or older who survive breast cancer, heart disease has now become the leading cause of death [2]. Cancer therapy-induced heart failure is particularly morbid, with an estimated 3.5 times greater risk of death compared to idiopathic cardiomyopathy [3]. The prognosis in these heart failure patients is even worse than those seen in ischemic cardiomyopathy patients [4]. In fact, chemotherapy-induced cardiotoxicity is the second leading cause of morbidity and mortality in cancer patients, with malignancy being the first [5]. The scope of the cardiotoxicity challenge has resulted in the emergence of a new field: cardio-oncology. Cardiotoxicity detected during cancer therapy often leads to a stoppage of treatment or a switch to a less effective option, negatively impacting outcomes. Better cardioprotective intervention strategies that do not interfere with therapy efficacy can thus improve the current treatment of cancer patients. However, the current knowledge gap limits the use of adjuvants that protect the cardiovascular system while preserving treatment efficacy, raising the urgent need for a deeper mechanistic understanding of cellular processes affected by cancer drugs and potential cardioprotective interventions.

### 1.2. Autophagy: A Well-Conserved Cellular Degradative and Recycling Process

**Figure 1:**
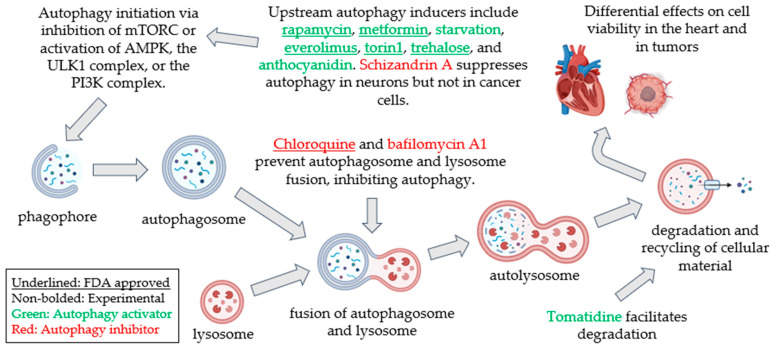
Autophagy modulations by genetic, pharmacological, or dietary means target specific steps of the autophagic pathway. Autophagy can be induced genetically by inhibitors of the mTORC1 and mTORC2 complexes, the activation of the AMP kinase pathway, and the downstream ULK1 and PI3K complexes. Pharmacological interventions such as rapamycin [14,15], everolimus [16], and torin1 [17] are robust mTOR inhibitors and activate autophagy. Dietary intervention by starvation [18] or pharmacologic metformin [19] induces autophagy via the mTOR and AMPK pathways. Trehalose inhibits glucose uptake, inducing starvation [20]. Schizandrin A induces autophagy and apoptosis in cancer cells [21] but suppresses autophagy during neuronal injury [22]. Downstream in the autophagic process, lysosomotropic agents such as chloroquine prevent autophagosome and lysosome fusion [23] and inhibit autophagy. Bafilomycin A1 inhibits autophagy by targeting the vacuolar-type H-ATPase (V-ATPase) proton pumps on lysosomes [24]. Tomatidine promotes lysosomal activity via the activation of transcription factor EB (TFEB) [25]. Autophagy modulation and inhibition differentially impact cell death and viability in the heart and in tumors.

A common pathway that differentially affects cardiovascular health and cancer is autophagy. Since the Nobel Prize was awarded in 2016 for the discovery of autophagy, many biological insights into the molecular and regulatory mechanisms of autophagy have been gained [6]. This review focuses on macroautophagy—hereafter referred to as autophagy—as other forms of autophagy such as microautophagy, chaperone-mediated autophagy, and substrate-specific autophagy are discussed in detail in other reviews [7,8,9]. Autophagy is a cellular homeostatic process conserved in virtually all mammalian cells to eliminate harmful stressors and recycle cellular constituents. Autophagy encompasses distinct stages: initiation, nucleation of the autophagosome, expansion and elongation of the autophagosome membrane, closure and fusion with the lysosome, and degradation of intravesicular products. Each stage has a subset of potential therapeutic targets for activating or inhibiting autophagy, achieved by genetic, pharmacological, or dietary interventions (Figure 1), providing potentially promising strategies for treating diverse human diseases [10,11]. During stress, dysfunctional organelles and proteins are sequestered into autophagosomes, trafficked to the lysosomes to form autophagolysosomes for degradation, and subsequently released into the cytoplasm for recycling. Autophagy is also intricately linked to cell death processes, including autophagy-mediated apoptosis (termed “autosis”), ferroptosis, and necroptosis, all of which are active areas of research to advance the treatment of cancer [12], as well as more recently in cardiovascular disease [13].

**Figure 2:**
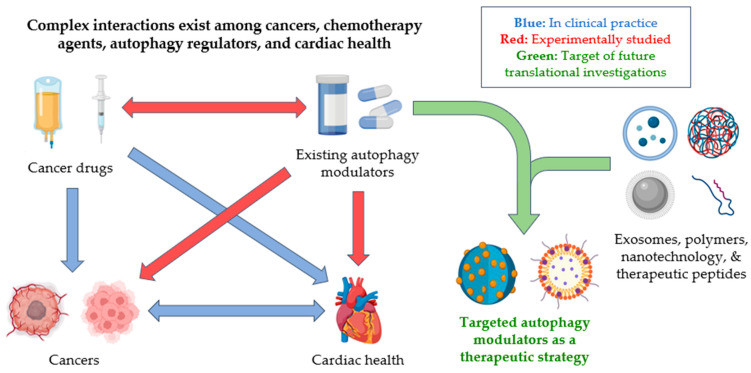
Cross-links between autophagy modulators, cancer treatments, cancer, and the heart. Current clinical practices in oncology and cardio-oncology focus on the interplay between cancer, its treatments, and their long-term effects on cardiac function. While autophagy has been shown to have promising cardioprotective effects in certain contexts, there is currently a lack of understanding regarding its effects on different cell types, especially under various disease and treatment conditions. Further investigations of autophagy’s mechanism and kinetics, as well as the creation of a translational cardio-oncology model, are required to realize its potential as a therapeutic strategy.

The autophagic process is dynamic [26,27]. The kinetics of autophagic flux, however, have not been characterized in disease states [28]. The biological impact of changes in autophagy on cell physiology and fate remains poorly characterized (Figure 2). The major limitations highlighted in this review are the following: (i) The lack of autophagy reporters that can track autophagy kinetics in real time [29]. Commonly deployed gold standards in autophagy detection, such as by fluorescent reporters of LC3, are not translatable to human clinical sample analysis. On the other hand, the biochemical analysis of autophagy biomarkers LC3, p62, and Beclin-1 can only accurately measure autophagic flux if repeated biopsy samples can be collected [30]. (ii) There are no suitable in vivo models that preserve the microenvironment, the heterogeneity of cell types in tissue, and cell–cell communication. (iii) Cardiotoxicity in cancer mouse models is still in its infancy. No translational model has been validated to investigate cardiotoxicity in large animal models, limiting the translation of the biological understanding gained at the bench to its impact in the human clinic. A multi-faceted interdisciplinary endeavor is thus needed to fully realize the potential of autophagy modulation to treat cancer and cardiovascular disease.

### 1.3. Autophagy Pathophysiology: Differential Impact of Autophagy Modulation on the Heart and Cancer

The biological impact of autophagy on cancer is relatively well studied. In the early stages of malignancy, defective autophagy promotes cancer initiation and progression as damaged organelles and proteins fail to be degraded, eliciting inflammation that modifies the tumor microenvironment and exacerbates cancer [31,32,33]. Paradoxically, in the later stages of malignancy, autophagy has been shown to promote tumorigenesis and metastasis by inducing neovascularization and other invasive properties including the secretion of pro-invasive cytokines, the detachment of metastatic cells, and the survival of cancer cells [34,35,36]. Excessive autophagy can either increase cancer cell death or increase unwanted chemoresistance [37,38]. Hence, autophagy may be a prognostic biomarker of cancer to better stage progression and tumorigenesis [39,40,41,42].

On the other hand, autophagy is an adaptive mechanism in the heart. The activation of cardioprotective autophagy and mitophagy in cardiomyocytes allows for the rapid degradation and recycling of intracellular stressors to maintain cell viability, and the effects of autophagy stimulation are well covered in previous reviews [43,44,45], demonstrating that the acute activation of autophagy exerts beneficial effects on cardiomyocytes in various forms of cardiomyopathy leading to heart failure. However, it remains controversial when and how autophagy modulation is safe as an adjuvant to minimize cardiovascular dysfunction in cancer patients.

### 1.4. Unrealized Therapeutic Potential of Autophagy Modulation to Simultaneously Treat Cancer and Cardiovascular Disease

Autophagy levels in cancer cells are highly dysregulated compared to those in non-cancerous cells [46]. Autophagy activation is likely to be beneficial in preventing the transformation of healthy cells into cancerous ones by clearing damaged organelles [47]. As tumors form and mature, autophagy may be activated in response to oxidative stress in the microenvironment to sustain tumor growth and metastasis [48]. Thus, it is unsurprising that autophagy inhibition by FDA-approved small molecules, such as chloroquine, aids in treating cancers at the bench and in clinical trials [49]. Many cancer treatments can also impact autophagy (Table 1). Paradoxically, treatments highly effective at mitigating cancer also activate autophagy [50]. Therefore, our understanding of the pathophysiological role of autophagy in cancer cells and the tumor response to cancer treatment remains incomplete.

Cancer drugs can induce the death of cancer cells via multiple autophagy-mediated apoptotic and non-apoptotic pathways, including autosis [72], ferroptosis [73], and necroptosis [74], which are emerging targets for novel cancer treatments. In fact, many drugs with efficacy against cancer have been shown to modulate autophagy, leading to the activation of cell death programming (Table 1). These same apoptotic and non-apoptotic pathways have also been linked to cardiovascular disease [75,76]. Accumulating evidence supports that treatments for HER2-positive breast cancer, including non-targeted chemotherapeutics, such as doxorubicin, and newer targeted cancer drugs, such as trastuzumab, can directly affect autophagy in cardiomyocytes and other cell types in the heart [77,78]. Autophagy dysregulation due to cancer drugs results in cardiomyopathy and heart failure, leading to systolic [55] or diastolic dysfunction [79], hypertension, and arrhythmia [80]. The mechanism underlying how autophagy dysregulation leads to cardiovascular dysfunction in the context of cancer and cancer treatment is, however, not well understood.

In the heart, the excessive up- or downregulation of autophagy can be detrimental to cells and impact cardiovascular health [81]. The heart is composed of a myriad of cell types, including contractile cardiomyocytes, endothelial cells, fibroblasts, immune cells, and others. The impact of autophagy levels on cell fate is cell type-dependent and disease-specific. In cardiomyocytes, autophagy is an adaptive mechanism that rapidly turns over intracellular stressors, such as damaged mitochondria or misfolded proteins, by recycling their constituents to enhance cell survival. In other words, autophagy plays an important role in maintaining cardiomyocyte homeostasis [82]. The activation of autophagy in cardiomyocytes has been reported to exert cardioprotective effects in a myriad of cardiovascular diseases, including cancer treatment-induced cardiotoxicity [83]. Cardiomyocyte autophagy activation through AMPK can reduce ROS levels, leading to the downregulation of ROS-induced NLRP3 inflammasome activation [84], a reported therapeutic target for doxorubicin-induced cardiotoxicity [85]. However, the activation of autophagy in coronary endothelial cells can induce endothelial progenitor cell apoptosis, inhibit cell proliferation and migration, and suppress reendothelialization [86], as seen in in vitro studies and in patients with coronary stents placed for atherosclerosis [87]. More recently, endothelial autophagy activation has been shown to attenuate the endothelial-to-mesenchymal transition [88], which in a rat model of chronic doxorubicin-induced cardiotoxicity activates the nuclear factor (NF)-κB pathway, leading to vascular injury and cardiac fibrosis [89]. Autophagy modulation can thus lead to further endothelial injury and unwanted inflammation. In cardiac fibroblasts, autophagy activation enhances the maturation of myofibroblasts and can regulate fibrosis to prevent adverse remodeling [90,91]. Yet, overstimulating fibroblast activation can lead to unwanted fibrosis. Taken together, a similar holistic approach [92] that leverages autophagy modulation can be promising to prevent or treat cardiovascular dysfunction during cancer treatment, but this needs to be carefully defined.

Much like in the case of the heart, tumors are composed of diverse cell types. Given the complex pathophysiological impact of autophagy on cancer and cardio-oncology, precision medicine approaches will likely be more effective, taking cancer stage, cancer type, and the cancer and cardiac responses to cancer treatment into consideration. Small molecules with robust autophagy-modulating effects have been studied extensively in preclinical cell and animal experiments and have been proposed to exert cardioprotective [93] or anti-cancer effects [94]. The signaling pathways or specific processes affected by autophagy modulators are often well established. For instance, rapamycin activates autophagy via the mTOR (mammalian target of rapamycin) pathway and has been shown to exert beneficial effects in cardiovascular diseases [95] as well as in cancer treatment [96]. Chloroquine, a well-established autophagy inhibitor that inhibits lysosomal fusion with autophagosomes, has been approved for clinical use in cancer treatments [49]. The impact of chloroquine on cardiotoxicity during cancer treatment, however, remains unknown.

A plethora of small molecule compounds capable of either activating or inhibiting autophagic processes are available and, in some cases, approved by the FDA. The established safety profile for human use certainly raises hope that the off-label use of drugs such as rapamycin, chloroquine, metformin, and statins as autophagy modulators can accelerate translation. However, concerns remain that autophagy modulation affects different cell types very differently and can often exert opposite effects from the desired outcome. The off-target effects of autophagy modulation thus require careful consideration when delivered systemically. This is currently a major limitation in advancing autophagy modulation to treat disease. Targeted modulation specific to the autophagy process or to specific cell types or organs is therefore sorely needed to leverage the full potential of autophagy as a therapy.

### 1.5. Non-Small Molecule Approaches to Achieve Targeted Autophagy Modulation

#### 1.5.1. Nanoparticulates

Polymers of nanoparticulate constructs can be used to enhance the delivery of autophagy-modulating peptides and enhance their bioavailability to specific cells or organs. Since the description of a well-established autophagy-activating cell-penetrating Tat-Beclin 1 peptide composed of the human immunodeficiency virus 1’s transduction domain (Tat) fused to a conserved 18-amino acid sequence of Beclin 1 [97], further peptide modification strategies, particularly a diversity-oriented stapling approach, have been reported [98]. This approach produces an array of chemically circularized (“stapled”) Beclin 1-derived peptides to facilitate efficient cell penetration without the Tat sequence, and their diverse structures allow the diversity-oriented screening [99] of stapled peptides to isolate bioactive autophagy inducers. The most potent stapled peptide DD5-o at a micromolar concentration exerts autophagy-activating and aggregate-clearing activities in vitro in a cellular model of Huntington’s disease and induces autophagy in the muscles of mice in vivo [98]. Polylactic acid (PLA) particles surface-adsorbed with Tat-Beclin exhibit enhanced hepatic uptake in mice in vivo to induce autophagy and reduce lipid accumulation in an in vitro model of non-alcoholic fatty liver disease [100]. This confirms that not only is the Tat-Beclin peptide delivered intracellularly but it is also subsequently released and maintains its autophagy stimulating effects.

The cell-specific targeting of autophagy modulation is also feasible utilizing cell-type-specific targeting peptides that bind to the intended surface receptors. Cancer cell targeting is commonly achieved by RGD (Arginine–Glycine–Asparagine) peptides. For instance, a study demonstrated the synthesis of melanin-like polydopamine nanoparticles modified with dual RGD (which targets the alphavbeta3 integrin receptor on tumors) and Beclin 1-derived peptides (binds to intracellular Class III phosphatidylinositol 3-kinase) to induce autophagy in cancer cells [101]. The use of nanocarriers to modulate autophagy allows the pharmacodynamics and pharmacokinetics of autophagy modulation to be controlled, leading to better bioavailability. This is a potentially attractive option for translation.

While cancer-targeting peptides recognize overexpressed cancer cell receptors, cardiac targeting can be achieved by Cardiac Targeting Peptides. These peptides bind to potassium voltage-gated channel subfamily H member 5 (Kcnh5) to target murine and human cardiomyocytes [102]. The cardiomyocyte targeting of autophagy-modulating molecules or peptides, however, has not been reported. The specificity of these peptides to deliver autophagy-modulating molecules to cardiomyocytes compared to cancer cells warrants further investigation.

Interestingly, various nanoparticles have been shown to directly affect the autophagy process. Cerium oxide nanoparticles protect against doxorubicin-induced cardiotoxicity by increasing autophagic flux, myocardial cardiac enzymes, and myocardial contractility [103]. Graphene oxide (GO) is an ultrathin nanomaterial that has been shown to be capable of stimulating or inhibiting autophagy in cancer cells [104]. This suggests that while nanoparticles may be an attractive translational tool to enhance autophagy modulation when delivered systemically, the biological effects of nanoparticulates on specific cell types need to be carefully evaluated to achieve the desired autophagy-modulating effects.

Immune cells have been engineered directly to specifically modulate autophagy. For instance, in mice, macrophages have been engineered to induce LC3-dependent autophagy to enhance the efficacy of a Bacille Calmette–Guérin (BCG) vaccine, commonly used against tuberculosis [105]. Autophagy activation increases the antigen presentation of macrophages in the fight against tuberculosis. In another study with healthy human volunteers, autophagy was specifically induced in vaccine-induced antigen-specific CD8+ T cells [106]. The study provided evidence for the importance of autophagy in vaccine immunogenicity in older humans and uncovered two novel drug targets (translation factor eIF5A and transcription factor TFEB) that may increase vaccination efficiency in the elderly population. These studies raise intriguing possibilities to enhance immunotherapy by leveraging autophagy modulation and to further investigate its effects on cancer treatment as well as its associated cardiotoxicity.

#### 1.5.2. Extracellular Vesicles

Naturally occurring circulating biomarkers, such as extracellular vesicles (EVs), are an emerging area of research in the diagnosis of cancer [107,108,109,110,111,112] and cardiotoxicity [113,114,115,116,117] and can also be engineered for therapeutic effects [118,119,120,121,122,123,124]. EVs are heterogeneous, nano-sized, double-membraned vesicles secreted endogenously by all cell types that can carry signaling molecules (e.g., lipids, nucleic acids, and proteins) and whole organelles [125]. EVs are important biomarkers of human disease that have also been explored as potential therapeutics. For instance, EVs from adipose-derived stromal cells were shown to contain miR-93-5p, which is responsible for inhibiting hypoxia-induced autophagy and inflammation and attenuates myocardial infarction in rats [126].

Additionally, EVs can modulate autophagy to protect tissues during periods of cellular stress [127]. Exosomes from induced pluripotent stem cell-derived cardiomyocytes promoted autophagosome production and autophagic flux, reduced apoptosis and fibrosis, and improved myocardial repair in mice [128]. Mesenchymal stem cell (MSC)-derived exosomes effectively protected hepatocytes against D-galactosamine and lipopolysaccharide (D-GalN/LPS)-induced damage via increasing levels of autophagic-related proteins (LC3B and Beclin-1) and the suppression of pro-apoptotic proteins [129]. Similar results were obtained in a rat model of spinal cord injury where the authors showed that neural stem cell-derived exosomes suppressed apoptosis and neuro-inflammation by inducing autophagy while reducing pro-inflammatory cytokine expression [130]. Exosomes from human MSCs also demonstrated the potential to attenuate ischemia–reperfusion injury by the inhibition of autophagy flux through the upregulation of mTORC1/p-4eBP1 [131]. However, the therapeutic potential of exosomes in preventing cardiac injury and the subsequent development of cardiomyopathy in cardio-oncology is not yet clear. Caution and further research are thus needed to harness the therapeutic potential of EVs to modulate autophagy in the target cell types.

More recently, autophagy has been implicated in the regulation of EVs [132,133,134]. Cargo sorting in EVs is achieved via autophagy machinery proteins [133]. Given that EV cargoes play an important role in their biological effect on recipient cells, it is important to understand the mechanism by which EV cargoes can specify the autophagy response, and how this response differs in cancer and cardiovascular cells under physiological and pathophysiological conditions.

Given that EVs are naturally occurring nanostructures, they have potential for translation. Further investigation into the impact of nanomaterials on the cardiovascular system and cancer will accelerate the translation of nanomedicine into the clinic as a novel class of biological molecules for targeted autophagy modulation.

#### 1.5.3. mRNA

mRNA can also modulate autophagy, potentially opening the door for future targeted therapeutic strategies. Site-specific mRNA delivery methods are an emerging area of targeting strategies with increased payload delivery efficiency. These include the use of organ- or tissue-specific lipid nanoparticles (LNPs) following local injection, as well as organ- or cell-specific LNPs after intravenous injection [135]. mRNA was also functionalized on nanoparticles for delivery in a translational mouse model [136]. The mRNA nanoparticle-mediated restoration of PTEN in PTEN-null tumors has also shown that autophagy can be specifically induced to trigger cell death-associated immune activation. These mRNA-mediated autophagy modulation approaches can potentially support novel immunotherapies. However, potential off-target effects on other organs need to be carefully studied to overcome similar challenges faced by other mRNA-based therapies.

#### 1.5.4. Other Strategies

More recently, cargo-specific autophagy has been explored to translate autophagy understanding into therapeutics. Autophagy-inhibiting peptides are useful in treating cancer [137]. Targeted peptides have been shown to maintain protein homeostasis and show specificity in modulating the proteosome pathway, which is distinct from macroautophagy [138]. The efficacy of these technologies is yet to be well tested in cancer and cardiovascular cells.

AceTAC degradation technology utilizes antigen–antibody interactions and autophagy to break down and remove intracellular stressors such as proteins [139]. Variations in this technology, such as AUTOTAC, utilize bifunctional small molecules to link p62 to specific targets, efficiently degrading selected proteins via autophagy [140]. More recently, the feasibility of the targeted degradation of organelles such as mitochondria, peroxisomes, and the endoplasmic reticulum in mammalian cells has been demonstrated [139]. Interestingly, cargo-specifying technology has also been explored to develop therapeutic strategies that “hijack” autophagy machinery to degrade specific cargoes. These areas of research may provide targeted autophagy therapeutics to tinker with the delicate balance in treating cardiotoxicity during cancer treatment.

In the era of artificial intelligence and big data, machine learning and system biological approaches have shed novel insights into autophagy modulation. Using a machine learning, cross-species workflow, eighteen small molecule autophagy modulators, including two novel mitophagy activators, were identified [141]. The authors further demonstrated that activating mitophagy was beneficial in mitigating memory loss and other disease pathologies in Alzheimer’s disease models of C. elegans and mice. A separate study using mathematical and computational modeling revealed that NRF2 (nuclear factor erythroid-related factor 2) interacts with the key autophagy signaling molecules AMPK and mTOR to fine-tune autophagy [142]. Autophagy induction by targeting AMPK, NRF2, and mTOR together can better adapt to dynamically changing environments in response to oxidative stress. Another study used mathematical modeling to predict and optimize drug dosing to achieve controlled autophagy modulation [143]. The authors demonstrated a generalizable strategy derived using computational methods to guide autophagy-based therapy selection with optimal drug scheduling.

### 1.6. Translational Models of Cardio-Oncology

To ensure the successful translation of the fundamental understanding of autophagy to improve the clinical management of human disease, cross-disciplinary collaboration among basic, translational, and clinical scientists is needed. The in vitro use of primary cardiomyocytes, whether of a human or large animal, however, remains a challenge as cardiomyocytes are terminally differentiated cells that are difficult to maintain in culture. Human-induced pluripotent stem cell-derived cardiomyocytes (iPSC-CMs) have been well validated for studying cardiotoxicity [144,145,146]. However, autophagy is sensitive to culturing conditions and has not been carefully characterized in iPSCs. It remains to be determined whether autophagy and the response to autophagy activation or inhibition in iPSC-CMs recapitulate what is seen in primary cardiomyocytes.

Primary cardiac slices, on the other hand, are 100–400 μm viable tissue slices generated from a living adult ventricular myocardium using a high-precision vibratome system [147,148]. The slices represent a suitable in vitro system that provides several major advantages over isolated cardiomyocytes: (i) the cardiovascular architecture and cell types are preserved; (ii) cardiac slices have been shown to survive in culture for one week; and (iii) the slices can be generated from all animal models as well as human tissue [147,148,149,150,151]. In addition, basal autophagy and the response to autophagy modulations in cardiac slices were recently validated in canine slices [147]. Cardiac slices are also amenable to co-culturing with cancer cells, 3D cancer organoids, and immune cells, creating a more realistic cancer and treatment setting more closely mimicking an in vivo scenario. This is critical in the context of cardio-oncology to understand the cardiac autophagy response to the presence of a tumor and the associated pro-inflammatory environment with or without the addition of chemotherapy and autophagy modulation. The cardiac response to chemotherapy with or without autophagy modulation is likely influenced by intercellular and inter-tissue or organ communications seen in the in vivo environment. Cardiac slices generated from healthy or pathological hearts isolated from large animals such as the canine provide a uniquely powerful in vitro model in drug development. The slice system thus presents an ideal preclinical evaluation tool for the high-throughput screening of drugs that may yield lead compounds to be further tested in canine models.

An appropriate translational animal model for in vivo cardio-oncology research remains lacking. Preclinical transgenic, humanized, and implanted tumor mouse models that are commonly used often do not faithfully recapitulate the complex and heterogeneous cancer phenotype in human patients [152,153]. This further limits the study of cancer treatment-induced cardiotoxicity in murine models [154,155]. Unlike mice, which do not spontaneously develop cancer, canines are particularly relevant in the study of human cancer and the cancer response to drugs as they develop spontaneous cancer much like humans and receive the same or similar cancer therapies and cancer drugs as humans. For example, it is well established that dogs receiving anthracycline develop cardiotoxicity manifesting as systolic dysfunction, heart failure, and arrhythmias, similar to human cancer patients receiving anthracycline therapy [156]. These canine patients also develop systemic hypertension in response to tyrosine kinase inhibitor treatments, similar to responses seen in human cancer patients [157]. Clinical trials in pet dogs undergoing cancer treatment thus present a uniquely suitable translational model to study responses associated with autophagy modulation, cancer treatment, and cardiotoxicity.

### 1.7. Autophagy Monitoring Technology

There is currently a lack of suitable biomarkers to follow the onset and progress of cardiotoxicity during cancer therapy. Given the pathophysiological significance of autophagy in both cancers and the heart, validating autophagy as a potentially viable prognostic biomarker in the context of cardio-oncology warrants further investigation. In healthy cells, basal autophagy involves the intricate intracellular trafficking of autophagic vesicles, which is highly dynamic and tightly regulated [33,158]. During disease states, the dysregulation of autophagy alters the kinetics of autophagic activity (referred to as autophagic flux). To accurately assess autophagic flux, autophagy must be studied longitudinally or measured at multiple time points. Fluorescent reporters of autophagy proteins such as the dual fluorescent LC3 (Light chain 3, a marker of autophagic structures destined for degradation and recycling) plasmid have allowed autophagic flux to be measured in real time in vitro [159,160,161]. Transgenic mice ubiquitously expressing genetic fluorescent reporters of autophagy are also well established for studying autophagy in vivo [149,162,163]. Engineering florescent autophagy reporter mice with cancer allows autophagy in cancer and cardiotoxicity to be studied in the in vivo environment.

The real-time monitoring of autophagy levels non-invasively in vivo was recently demonstrated [164]. Autophagy-detecting nanoparticles (ADNs), rationally designed to translocate into cells efficiently, co-traffic with early autophagosomes to lysosomes, allowing autophagy to be imaged with high sensitivity and specificity, and are quantitative of autophagic flux [164]. Probe-based autophagy imaging using injectable nanoprobes such as ADNs emits activatable near-infrared fluorescence, allowing autophagy to be detected and quantified in cells in vitro and in small animal models non-invasively in vivo [164]. An ADN injected intravenously reaches all major organs, allowing longitudinal and systemic autophagy monitoring to optimize autophagy modulation in the preclinical context. In other words, this allows a holistic approach to guide autophagy modulation that maintains homeostasis in the heart while maintaining cancer treatment efficacy.

Additionally, the ADN is biocompatible, synthesized based on FDA-approved ferumoxytol, a drug used to treat amenia. Ferumoxytol in humans has a blood half-life of 14 to 21 h and is eliminated via the hepatic and splenic routes [165]. The ADN produces activatable magnetic resonance signals, detectable by non-invasive medical imaging modalities such as magnetic resonance imaging (MRI). The ADN-enhanced imaging of autophagy needs further validation in translational animal models. Given the biomedical significance of autophagy as a viable biomarker, further developing autophagy imaging translational to the clinic is a highly novel and worthwhile endeavor.

## 2. Conclusions and Future Outlooks

Autophagy modulation as a therapeutic strategy has tremendous potential, but obstacles remain for clinical adaptation. The field of cardio-oncology presents particularly unique challenges as well as opportunities to leverage the power of autophagy biology and innovations in autophagy modulation to revolutionize the treatment of cancer and cancer therapy-associated cardiotoxicity. A deeper biological understanding is needed to untangle the differential impact of autophagy activation or inhibition on cardiovascular health while preserving or, in ideal cases, enhancing cancer therapy efficacy.

**Figure 3:**
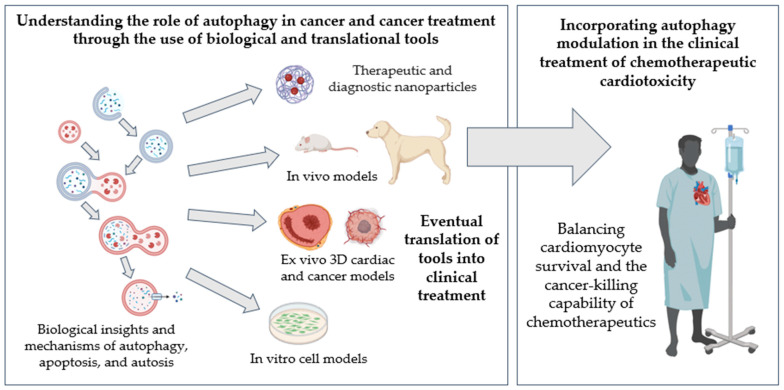
Overcoming the translational hurdles of autophagy modulation in cardio-oncology. The effects of chemotherapeutics, autophagy response to cancer drugs, and autophagy modulation must be investigated simultaneously in the heart and tumors. Various interdisciplinary approaches can be taken, including cell biology, 3D ex vivo tissue models, and in vivo preclinical mouse and translational canine cancer models. Specifically, pet dogs that spontaneously develop cancer and receive subsequent cancer therapy can develop cardiovascular dysfunction, thus serving as a uniquely valuable translational model. The cross-species platform, combined with diagnostic and therapeutic nanotechnology, can successfully impact the clinical management of millions of cancer patients and survivors who face an increased yet unpredictable risk of developing cardiovascular dysfunction, reducing unwanted cardiotoxicity, enhancing cancer treatment efficacy, and improving clinical outcomes.

This review highlights non-small molecule approaches to modulate autophagy, including engineered peptides that target specific autophagy-regulating proteins or nanoformulations capable of increasing bioavailability to the target cell type or organ. Nanomedicine has been an active area of research for cancer therapy [166,167,168] and may be a novel approach to mitigating cardiotoxicity by targeting autophagy modulation [169,170,171]. Nanotechnology further allows the probe-based monitoring of autophagy levels non-invasively in vivo [164]. Moreover, novel translation model systems are now available for new cancer drug testing to bridge preclinical investigations to the clinical setting. These highlights are summarized in Figure 3.

Future research directions to overcome the hurdles of translating autophagy modulation into clinical practice are sevenfold: (a) Biomarker discovery, likely from non-coding RNAs [172] or multi-omics studies [173], can identify specific biomarkers that could predict the effectiveness of autophagy modulation to guide cancer treatment while preventing cardiotoxicity. (b) The further development of molecular imaging of autophagy non-invasively in vivo can open novel portals into dynamic autophagy changes in the heart and tumors during cancer treatment. (c) The gain of a deeper mechanistic and pharmacological understanding in biological systems or the optimization of drug delivery methodologies can minimize the off-target effects of both small-molecule and non-small-molecule autophagy modulators. (d) Drug screening platforms that are amenable for translation must be enhanced. (e) Finally, multidisciplinary approaches through collaborations among basic scientists, translational researchers, and clinical cardiologists and oncologists must be integrated. Further autophagy research promises to treat cardiovascular disease and cancer with precision and provide real-time monitoring of treatment responses to improve outcomes. The research endeavor will not only impact the field of cardio-oncology, but it will also improve the treatment of a plethora of human diseases where autophagy dysregulation plays a role.

## Figures and Tables

**Table 1:** Differential effects of cancer drugs on autophagy and associated processes in the heart and tumors.

Biological Effects	Cancer Drugs’ Impact on the Heart	Cancer Drugs’ Impact on Tumors	Biological Effects Seen in Untreated Tumors
Autophagy	Reduced by doxorubicin [51] Induced by cisplatin [52]	Reduced by doxorubicin [53] Induced by cisplatin [54]	Reduced during tumor initiation; induced during malignancy [36,37]
Autosis	Induced by doxorubicin [55]	Induced by cisplatin [56] Induced by methotrexate [57]	Induced with increased autophagy levels [58]
Apoptosis	Induced by doxorubicin [59] Induced by cisplatin [60] Induced by methotrexate [61]	Induced by doxorubicin [62] Induced by cisplatin [63] Induced by methotrexate [64]	Reduced in tumors [65]
Oxidative Stress	Induced by doxorubicin [66] Induced by cisplatin [67]	Induced by doxorubicin [68] Induced by cisplatin [69] Induced by methotrexate [70]	Induced in tumors [71]

## Data Availability

Not applicable.

## Abbreviations

MTOR	mammalian target of rapamycin
AMPK	AMP-activated protein kinase
FDA	U.S. Food and Drug Administration
V-ATPase	vacuolar-type H+-ATPase
TFEB	transcription factor EB
LC3	light chain 3
TAT	transactivator of transcription
PLA	polylactic acid particles
RGD	Arginine–Glycine–Asparagine peptide
Kcnh5	potassium voltage-gated channel subfamily H member 5
GO	Graphene oxide
BCG	Bacille Calmette–Guérin
EIF5A	eukaryotic translation initiation factor 5A
EV	extracellular vesicle
D-GalN	D-galactosamine
LPS	lipopolysaccharide
MSC	mesenchymal stem cell
p-4eBP1	phosphorylated translation initiation factor 4E-binding protein 1
LNP	lipid nanoparticle
PTEN	phosphatase and tensin homolog
AceTAC	autophagy receptor-inspired targeting chimera
AUTOTAC	autophagy-targeting chimera
NRF2	nuclear factor erythroid-related factor 2
iPSC-CMs	human-induced pluripotent stem cell-derived cardiomyocytes
ADN	autophagy-detecting nanoparticle
MRI	magnetic resonance imaging
